# The quantitative evaluation of subjective satisfaction with digital media in L2 acquisition in younger adults: A study from Europe, Asia, and Latin America

**DOI:** 10.3389/fpsyg.2022.946187

**Published:** 2022-11-17

**Authors:** Marcel Pikhart, Blanka Klimova, Liqaa Habeb Al-Obaydi, Szymon Dziuba, Anna Cierniak-Emerych

**Affiliations:** ^1^Department of Applied Linguistics, Faculty of Informatics and Management, University of Hradec Králové, Hradec Králové, Czechia; ^2^English Department, College of Education for Human Sciences, University of Diyala, Baqubah, Iraq; ^3^Faculty of Business and Management, Wroclaw University of Economics and Business, Wroclaw, Poland

**Keywords:** L2 acquisition, foreign language learning, ESL, EFL, digital media, psycholinguistics, applied linguistics

## Abstract

Foreign language learning has recently been transferred into an online or hybrid mode and this has brought many challenges for both the teachers and the students. Thus, the purpose of this study is to explore students’ subjective satisfaction with the use of digital media in their L2 acquisition conducted online, as well as to provide specific recommendations for meeting students’ needs in digital media L2 instruction. This is large-scale comparative research conducted in the Czech Republic, Poland, Romania, Iraq, and Malaysia. The data were collected through an online questionnaire in May, June, and July 2021 in the given countries. The findings reveal that students’ subjective satisfaction that is related to students’ attitudes toward the online learning process, the general usefulness of language, the role of the teacher, and the matters that affect the general process of teaching and learning all gained the positive answers. Whereas the items that are related to students’ subjective satisfaction toward language skills, digital-based reading, the effectiveness of online education over face-to-face, and communicating with teachers and peers *via* social media are all gained negative results. These results need further analysis but they can be an impetus for much larger research and further implications to optimize L2 acquisition.

## Introduction

The impact of digital media and their use in various aspects of human life is undisputed, mostly in the last decade and in the younger generation, i.e., Generation Z, iGen, or the so-called Zoomers (Barber, 2020). This is the first generation that has grown up with access to the Internet and portable digital technology from a young age. The impact of digital media on their free time activities, entertainment choice, communication, and interaction has already been well-documented (Greve et al., 2022). However, the effect of digital media on the second language (L2) acquisition still needs an in-depth investigation. Moreover, university education has been moved online due to the COVID-19 pandemic, which has affected the whole process of teaching and learning (Education in a Pandemic, 2021; The Disparate Impacts of COVID-19 on America’s Students, online). Technology has become an inseparable part of everyday instruction and student learning. The course contents had to be redefined and remodeled in order to meet students’ online learning needs and the so much desired quality of teaching so that students could achieve their learning goals and thus be satisfied with this new form of instruction.

In fact, students’ satisfaction with the quality of digital media instruction is directly connected with their improved learning performance and perceived usefulness of these technologies. There are, of course, several factors or determinants, which play a significant role in their satisfaction with digital media. The findings of research studies reveal that one of the crucial factors is quality teaching, which is ensured by a teacher. Gopal et al. (2021) maintain that teachers conducting their instruction online have to understand students’ psychology in order to be able to properly deliver the course content. In addition, they have to meet students’ learning needs, design understandable course content, and provide prompt feedback to their students. Likewise, Keržič et al. (2021) report that the quality online teaching is associated with teacher’s active role and attitude to online teaching, preparation of regular assignments and openness to the students’ suggestions. She et al. (2021) expand that students’ satisfaction with online learning is affected by their interactions. The authors claim that the learners who interact more frequently during their online classes exhibit higher levels of learning satisfaction. Their findings also reveal that the learners who are more engaged in their studies are also more satisfied with online learning than those who are less engaged. Similar findings were highlighted by Kartika et al. (2021), who report that enhanced course design, teacher quality, interaction, and technology quality improve students’ satisfaction with online learning. Although interactivity as an important determinant appears in another study by Hettiarachchi et al. (2021), the authors consider the key factor of students’ satisfaction with the online mode of instruction perceived learner motivation since in online learning students perform self-regulated learning and they need to be responsible learners and thus self-motivated to do the assigned tasks. Furthermore, the authors also highlight perceived challenges of the online mode, such as isolation or problems with technological tools.

The findings described above about students’ satisfaction with digital media instruction do not differ from students’ satisfaction with digital media foreign language (FL) instruction. Pham and Nguyen (2021) in their study show that interaction with peers, the quality of course content, and the teacher’s pedagogical role as a provider of meaningful feedback, and self-regulation are good predictors of students’ satisfaction with online FL classes. In addition, Yang and Yang (2021) point out that such an online mode of FL learning is more suitable for learners with a higher level of FL proficiency. Their results also reveal that students had a more reserved attitude toward online FL instruction since they felt that their FL learning was affected by decreasing motivation, greater distraction, lack of actual interaction, peer pressure, teacher monitoring, and practitioner’s fatigue. As Tratnik et al. (2019) also indicate, the key drivers for students’ satisfaction with digital media foreign language (FL) instruction include course delivery, course quality, students’ expectations, motivation, student–student interaction, and perceived level of knowledge acquisition.

Therefore, the purpose of this study is to explore students’ subjective satisfaction with the use of digital media in their L2 acquisition conducted online and to confirm or deny the present findings, as well as to provide specific recommendations for meeting students’ needs in digital media FL instruction.

The research questions were formulated to provide an answer to the aforementioned issues and are as follows:

1. Are present university students satisfied with acquiring L2 only online?
2. Which factors affect students’ subjective satisfaction with The Use of digital media In their L2 acquisition?

## Methodology

The data were collected by a questionnaire submitted *via* Google Forms to the students of the universities as follows:

1. Faculty of Informatics and Management of the University of Hradec Kralove, Czech Republic (*n* = 113),
2. Faculty of Business and Management of the Wroclaw University of Economics and Business, Poland (*n* = 120)
3. Faculty of Law, Dimitrie Cantemir Christian University, Romania (*n* = 102)
4. English Department, College of Education for Human Sciences, University of Diyala, Iraq (*n* = 394)
5. Language Academy, Universiti Teknologi Malaysia Johor Bahru, Malaysia (*n* = 70) and Universiti Teknologi Malaysia Kuala Lumpur, Malaysia (*n* = 133), total for Malaysia *n* = 203
6. Universidad Nacional Autónoma de México, Mexico (*n* = 209)

All universities are well-established universities in their respective country and they are state universities with a sufficient and comparable IT infrastructure. The choice of the universities was random and based on the initiative of a scholar who responded to our plea for the data collection. The total number of respondents was 1,110 from all the given countries. Around a third of responses were obtained from Europe, a third from Iraq, and the last third from south-east Asia. The data were collected in May, June, and July 2021. All the respondents agreed with taking part in the questionnaire survey and the Ethics Committee of the University of Hradec Kralove approved this research (no. 2/2021). There was no experiment or intervention involved, and no personal data about the participants were collected. The only identification of the respondent was the time stamp when the questionnaire was submitted. GDPR was fully observed and no ethical issues were raised as all the data were only collected anonymously online without any intervention or experiment conducted on the participants.

The research questionnaire consisted of two parts:

- a respondent data section, including five sociodemographic questions, and a filter question included in the introduction;
- the main part, which contained 19 questions with answers expressed on a 5-point Likert scale (Strongly Disagree, Disagree, Undecided, Agree, Strongly Agree) and one multiple-choice question (Which language skills do you think are good to practice online?)

In this survey, we aimed to make a statistical analysis of data of online language learning for six different nationalities: Czech, Polish, Romanian, Malaysian, Mexican, and Iraqi.

The following abbreviations were used:

- CZ—Czech nationality
- IRQ—Iraqi nationality
- MAS—Malaysian nationality
- MEX—Mexican nationality
- POL—Polish nationality
- ROU—Romanian nationality

In the case of continuous data, the following descriptive statistics were used to describe the characteristics of the study group: mean, median, standard deviation (SD), the values of the first and third quartiles (IQR), and range: in the case of questions with responses on a 5-point Likert scale, the grading of 1–5 was adopted for comparisons (with 1 meaning “Strongly disagree” and 5 meaning “Strongly agree”). For categorical data, corresponding to the original Likert scale responses, the frequency distribution of each response was presented using the sizes of each category and their distribution expressed as percentages.

For parameters that did not have normal distributions and variables with ordinal scale, the consequence was the choice of appropriate methods of a statistical analysis based on non-parametric tests. Pearson’s chi-squared test was used to compare the relationships between two or more groups of observations. The Kruskal–Wallis test was used if data were transformed to ranks derived from the from 1 to 5 points given to the answers to the questions. The Kendall’s tau correlation coefficient, which is a measure of monotonic statistical relationship between the variables, was used to examine the presence of monotonic relationships between ordinal variables, as in the case of questionnaires using Likert scale scores.

In order to verify the internal consistency of the questionnaire design used in this study, a reliability analysis was conducted using the Cronbach’s alpha reliability coefficient, which measures the ratio of the variance of individual items in the questionnaire to the variance of the entire scale. To put it simply, the aim is to ensure that respondents’ answers to individual questions have relatively little variation, but at the same time, respondents’ answers to individual questions should be relatively varied (so that every respondent does not answer the same way), which indicates a natural variation of the phenomenon. The rule of thumb here is that the higher the correlation between answers to individual questions is, the higher the value of the coefficient it can achieve. Its value can be arbitrarily small and at most equal to 1. A score above 70% is considered satisfactory and a score as low as 60% is considered acceptable.

The level of significance level was set at *p* = 0.05, also indicating statistically significant results for levels of *p* = 0.01 and *p* = 0.001. The *p*-values indicating a statistically significant result are highlighted in bold. For values smaller than 0.001, the notation *p* < 0.001 was always used. All calculations and graphs were processed using the statistical software package R (version 4.0.2), and structural modeling was additionally performed using the Lavaan software package (the latest available version 0.6).

### Questionnaire reliability

Cronbach’s alpha was used to verify the reliability of the questionnaire, and it was also examined how the extraction of individual questions would change this index. The analysis showed that the questionnaire with the full set of questions and after extracting one of them was characterized by a good level of reliability (Cronbach’s alpha exceeded 0.7 in each case).

## Results

### General demographic data

Basic descriptive demographic characteristics below provide country, age, nationality, gender, time spent in front of the computer on weekdays, online foreign language courses with a teacher each week during the term, and the number of languages.

Thus, the basic descriptive characteristics of the research sample was as follows: CZ 10.2% (*N* = 113), IRQ 35.5% (*N* = 394), MAS_JB 6.3% (*N* = 70), MAS_KL 12% (*N* = 133), MEX 16% (*N* = 178), POL 10.8% (*N* = 120), ROU 9.2% (*N* = 102). Regarding the age structure of the research sample as the respondents were university students, the mean was 21.39 years, and median 21 years. Regarding the gender, the number of females was nearly twice as high than the number of males since there were 64.1% (*N* = 712) of females and 33.5% (*N* = 372) of males, 2.3% (*N* = 26) did not disclose their gender.

Furthermore, the statistical tests revealed significant differences between nationalities and age [years] (*p* < 0.001, Kruskal–Wallis test), age categories (*p* < 0.001, chi-square test), gender of respondents, and the number of languages (*p* < 0.001, Pearson’s chi-square). The respondents were mainly young people, which is proved by the average age of around 21 years for each nationality, with the median exceeding 21 years only for the Romanian nationality. The respondents were very young people aged 19–25 years in the Czech population and 18–29 years in Poland, whereas there was a much wider range of age in Iraq (15 and 40 years), Romania (19 and 53 years), and Mexico (19 and 50 years). The majority of respondents were women in Iraq (75.7%, *N* = 289), Malaysia (68%, *N* = 138), and Mexico (61.2%, *N* = 109). In Poland, women accounted for 50.5% of the respondents (*N* = 56), whereas in Romania, the percentages of women and men were similar and amounted to 49.5% (*N* = 50) and 48.5% (*N* = 49), respectively. Furthermore, in the Czech Republic, the percentage of women was 48.2% (*N* = 53), while the percentage of men was 50.9% (*N* = 56). The respondents most often learned one language online. This concerns people from countries such as Iraq (96.6%, *N* = 369), Czech Republic (90%, *N* = 99), Mexico (74.2%, *N* = 132), Malaysia (72.4%, *N* = 147), and Romania (70.3%, *N* = 71). Only Polish respondents indicated two foreign languages most frequently (93.7%, *N* = 104).

### Time spent at a digital tool and language learned

The initial part of the survey also identified the volume of time spent in front of the screen. Up to 2 h a day 11.1% (*N* = 123), 2 to 6 h a day 27.2% (*N* = 302), 6 to 12 h a day 41.7% (*N* = 463), 12 to 16 h a day 0% (*N* = 0), over 16 h a day 5.4% (*N* = 60), and 14.6% (*N* = 162) did not answer this question.

The results indicate that the most frequently taught foreign language is English 71.6% (*N* = 947), followed by German 5.4% (*N* = 72), and Japanese 5.1% (*N* = 68). The other languages with results higher than one are Arabic 3.8% (*N* = 50), French 3.8% (*N* = 50), Spanish 3.7% (*N* = 49) and Mari 1.3% (*N* = 17). There was a statistically significant difference between the selected language and nationality (*p* < 0.001, Pearson’s chi-square test). English was the most common language chosen in Czech Republic (90.2%, *N* = 110), Iraq (92.5%, *N* = 102), Mexico (78.5%, *N* = 172), Romania (69.2%, *N* = 83), and Poland (50.7%, *N* = 111). In contrast, among Malaysians, English was the language of choice for 43.4% of respondents (*N* = 102), and Japanese was the next most common language (24.7%, *N* = 58).

The conventional presentation of the frequency distribution of responses (the details are to be found in Table 1 which is provided in an Appendix) is analyzed and summarized below.

### Student’s preferences

On a positive note the vast majority of the respondents [strongly agree 35.6% (*N* = 283)], agree 53.3% (*N* = 424) like learning foreign languages, i.e., they are motivated and they feel there is a good sense in learning them. The vast majority realizes the importance of foreign languages for their future and they clearly expressed that in one of the statements of the questionnaire [“*I think that foreign languages will be useful in my future work*”—strongly agreed 60.1% (*N* = 478), agreed 32.5% (*N* = 259)].

A very similar situation is when they were asked about their preference regarding printed vs. electronic materials they are given by the teachers and they have to use. 19.3% (*N* = 154) strongly agreed, 34.2% (*N* = 272) agreed, and 23.5% (*N* = 187) were undecided. Later in the questionnaire, there was the same statement again formulated slightly differently (“*Electronic texts are better for me than printed books*”) to verify their answer to the previous question and the responses were very similar.

Regarding the format of the meetings, i.e., in-class vs. online, there was a very strong preference of in-class teaching over online. The statement “*I prefer studying with a teacher in a classroom rather than online*” generated 24.2% (*N* = 193) answers strongly agree and 40.5% (*N* = 322) agreed, which created a vast majority of the respondents who clearly expressed their preference toward on-site classes. There were several reasons expressed in the questionnaire. One of the major ones was the lack of social contact with peers [“*I miss meeting my friends when classes are online*”—strongly agreed 33% (*N* = 263), agreed 41.3% (*N* = 329)]. The distribution of the answers to the statement “*I find online classes more convenient than in-class learning*” was very balanced and it indicates that some respondents consider the benefits of not commuting, or staying at home in their well-known environment, which indicated that it is very subjective when the convenience is considered.

### Student’s subjective evaluation of the efficiency of online classes

A few questions of the questionnaire focused on the respondents’ subjective evaluation of the efficiency of online classes. The idea behind this was not to evaluate a specific teacher or their particular class but it was rather to look for a general evaluation of the educational process when conducted online. The statements like “*I find online classes more effective than traditional classes with a teacher*” and “*I believe that online language learning is more effective than traditional classroom teaching*” generated very similar results. The respondents generally do not consider online learning much more efficient. It is obvious they accepted this was one of the ways of teaching but they felt that not the most efficient. And this happens in the context of a well-prepared teacher as the vast majority agreed or strongly agreed with the statement that “*I think my language teacher is well prepared to provide online instruction*” [strongly agreed, 27.8% (*N* = 221) and agreed 53.6% (*N* = 427)]. Moreover, they also positively evaluated that “*I get enough feedback from the online teacher while learning the language*” with strongly agreed 10.6% (*N* = 84) and agreed 54.6% (*N* = 435).

One of the biggest drawbacks of online learning subjectively perceived by the respondents was the increase in screen time. As they all belong to Gen Z, which is technologically savvy and spends a big amount of time in from of their screen for entertainment, when looking for information, and for communication with their friends and family, they strongly acknowledged that adding another aspect of their life, i.e., education, on the screen is not the best choice. It is very obvious in their reaction to the statement “*I think we spend too much time in front of the computer*.” 39.9% (*N* = 318) strongly agreed with it and 41% (*N* = 326) agreed. However, the distribution of the answer to the statement “*I do not mind having my camera on during an online foreign language class*” was rather balanced, some respondents agreed and a similar number disagreed, which indicates that even if we are dealing with Gen Z they still want to keep privacy and many of them do not feel comfortable when their private zone is challenged. They are also aware of the potential risks related to the breach of private data and they want to protect them as much as possible.

Most respondents in all analyzed countries chose the answer “*Agree*” and “*Strongly agree*” as a response to the question of whether they liked learning foreign languages. Respondents from the Czech Republic, Iraq, Malaysia, and Poland most often indicated that they preferred printed materials to electronic ones. Furthermore, respondents from Mexico and Romania most often indicated that they had no opinion on the subject. The highest percentage of those who prefer in-class learning with a teacher to online learning gave the “*Agree*” response, regardless of the country surveyed. Respondents overwhelmingly agreed with the statement “*I miss meeting my friends when classes are online*,” except in Romania, where there was the highest percentage of people who disagreed. Online classes are more convenient than in-class learning, especially for people from Romania, followed by Poland, and the Czech Republic. In Iraq, there was the same percentage of people who answered “*Agree*” and “*Disagree*.” In contrast, Malaysian and Mexican respondents most frequently selected “*Undecided*” and “*Disagree*” responses. Contact *via* emails or e-learning platforms with the teacher is not better than a real meeting for respondents from the Czech Republic, Iraq, Mexico, and Poland. For those from Romania, email or e-learning platforms contact with the teacher was better than a real meeting more often than for those from other countries.

The highest percentage of people from the Czech Republic and Romania did not have an opinion on whether online classes are more effective than traditional classes with a teacher, whereas, in other countries, the highest percentage of people disagreed with this statement. Furthermore, people from all the countries included in the survey unanimously believed that foreign languages will be useful in their future work. People from the Czech Republic, Iraq, Mexico, and Poland are most likely to disagree that electronic texts are better for them than printed books, while people from Malaysia and Romania were undecided. Apart from respondents from Romania, the respondents did not agree that online language learning is more effective than traditional classroom teaching. Respondents, regardless of the country, did not worry and trust themselves when learning languages online and believed that their language teacher was well prepared to provide online instruction (highest percentage of “*Agree*” responses).

Most people from the Czech Republic and Iraq had no problem having the camera on during class, whereas the opposite was true for the other countries. Furthermore, most people other than those from the Czech Republic found learning a foreign language online to be very interactive. Respondents from all countries agreed that learning a foreign language while studying online improved their skills. Respondents from Iraq, Malaysia, Mexico, and Poland stated that they liked using a virtual environment to learn a foreign language. “*I receive enough feedback from my online teacher while learning the language*”—this statement supports the largest percentage of respondents, most pronounced in those from Poland and Romania. However, for most respondents, face-to-face classes are better than those online, with the exception being those from Romania. Respondents from all countries believe they spend too much time in front of the computer.

The second way of presenting the results was to convert the responses into a scale of variables with numerical values and present descriptive statistics: arithmetic mean, standard deviation (SD), median, interquartile range (IQR), and the range of values of the responses for such data, as shown in Appendix Table 2, which confirms the information in Appendix Table 1.

## Discussion

The results of this study reveal that the respondents, in all countries, agreed on most of the items in the questionnaire except for the following; “*Contact with teachers via e-mails or e-learning platforms is better for me than a real meeting*,” “*I find online classes more effective than traditional classes with a teacher*,” “*Electronic texts are better for me than printed books*,” “*I believe that online language learning is more effective than traditional classroom teaching*,” “*I do not mind having my camera on during an online foreign language class*,” and “*I prefer online learning to face-to-face teaching*.” These results can be summarized as follows: the student-teacher interaction is not enhanced by digital learning, the performance in the language skills and sub-skills is performed subjectively weaker, the efficiency of online education is also seen as lower than traditional education. The digital materials and online language learning are thus not preferred over traditional ones. These items represent the main subjective viewpoints of students in online education specifically in the L2 acquisition context and they have to be taken into consideration very systematically as they present a serious drawback to digital learning. If the users of these technologies see them primarily as not efficient to a certain extent, it can cause lower motivation and dissatisfaction in the first place as all these items related directly to students’ subjective satisfaction (Baber, 2020; Baber, 2021; Jiang et al., 2021).

On the contrary, the items that gained acceptance from most respondents were as follows: “*I like learning foreign languages*,” “*I prefer printed to electronic materials*,” “*I prefer studying with a teacher in a classroom rather than online*,” “*I miss meeting my friends when classes are online*,” “*I think that foreign languages will be useful in my future work*,” “*I do not worry and I trust myself when I learn languages online*,” “*I think my language teacher is well prepared to provide online instruction*,” “*I find that learning a foreign language online is very interactive*,” “*I believe that I am improving my foreign language skills while learning online*,” “*I like the use of the virtual environment for learning a foreign language*,” “*I get enough feedback from the online teacher while learning the language*,” and “*I think we spend too much time in front of the computer*.” These results can be summarized as follows: the students’ attitudes to printed materials were higher than to digital media, and there was a clear preference of face to face teaching as they were missing social interaction. Naturally, the majority of the participants acknowledged the benefits of the English language for their future work. However, many of the participants expressed their concern with the increased screen time while learning in front of their computers. One item (“*I find online classes more convenient than in-class learning*”) gained relatively equal agreement and disagreement by the participants. And the last item that gained approximately equal results with little agreement than disagreement was “*I do not mind having my camera on during an online foreign language class*.” The findings of the survey clearly show that the items related to students’ attitudes toward the learning process, the usefulness of language, the role of the teacher, and the matters that affect the general process of teaching and learning all gained positive answers, such as agree or strongly agree. A similar trend is described by many researchers who show general acceptance of students despite the existence of some challenges (Adedoyin and Soykan, 2020; Alahmadi and Alraddadi, 2020; Selvanathan et al., 2020; Shahzad et al., 2020; Derakhshan, 2021; Ta'amneh, 2021; Zeng and Wang, 2021). Despite the fact that there is some measurable variance in students’ responses, the results of this study can be generalized since they represent the current situation of students all over the world as they were covered by this survey comprising 1,110 EFL college students in six countries.

The results described above provide unambiguous answers to the research questions stated in the introductory part. Thus, it is obvious that however much students like learning a foreign language and they perceive its learning significant for their future professions, they are not satisfied with acquiring L2 online. They clearly state that they prefer face-to-face classes to their online counterparts. This is in line with the findings of other research, e.g., Ahsan et al. (2021), Al-Mawee et al. (2021), or Al-Nofaie (2021) whose students also found classroom milieu more suitable and satisfactory compared to the online mode of teaching though some studies conclude that their students gained satisfactions in both face-to-face and online environment (Coetzee and Oosthuizen, 2012; El-Sayad et al., 2021). One of the reasons is a lack of social interaction and in the present study also too much time spent in front of the screen, the preference for the printed materials in which students can highlight the unknown words and phrases or those they want to remember and retain. In addition, Kerzic et al. (2021) explain that students respond better to diversity in learning formats than just to one, in this case, the online format. Furthermore, FLL is based on the communicative language teaching approach that aims to achieve communicative rather than linguistic competence through learner interaction. And it is an immediate interaction, which the online environment cannot provide at its best.

As far as the factors that affect students’ subjective satisfaction with the use of digital media in their L2 acquisition are concerned, the results of this study clearly highlight that the main positive variables include the importance of FL learning for their future profession, teacher’s role, students’ self-regulatory learning/motivation, and perceived academic achievements in acquiring L2, which again confirms the results of Gopal et al. (2021), Kerzic et al. (2021), or Pham and Nguyen (2021). In fact, all these factors are typical of regular face-to-face FL classes and they are crucial for the successful mastering of any FL. As Gopal et al. (2021) maintain, *when the expectations of the students are achieved, then it leads to a higher satisfaction level of the student*.

The limitations of this study might be its one-sided focus on quantitative research. The authors are fully aware of this fact and they plan to publish another original study on qualitative findings, which have been also collected. However, due to the big amount of the collected data and their interpretation, they decided to split the existing findings into two research studies.

## Conclusion

The results of this study provide specific recommendations in order to meet specific students’ needs in digital media L2 acquisition. The findings indicate that L2 should be done ideally in a traditional way, i.e., in the classroom. However, if this is not possible, authors believe that a blended learning approach (i.e., a combination of the face-to-face teaching and online learning) can be implemented. And the teacher should try to support them in their online mode as much as possible through different communication tools, as well as through his/her ongoing feedback. In addition, a variety of approaches to L2 acquisition are required to enable students to be exposed to L2 acquisition in many different ways to be able to retain its authentic language structures and language skills, as well as to keep them motivated to study a foreign language in this way. Furthermore, in this process of L2 acquisition, the key role is played by the teacher who should exploit his/her subject, pedagogical, and ICT knowledge in order to satisfy students’ needs in digital media multidimensional FL class.

Foreign language learning, especially EFL, covers a wide area of education that has recently gained priority as a result of the globalized world, multinational corporations, and cross-cultural teams. As a result, any improvement in relation to online education whether in students’ subjective satisfaction or in subjective feelings of the usefulness of digital media in EFL at any level, from elementary schools to high schools and universities, will be beneficial and will potentially bring many advantages related to an improved motivation of both teachers and students. It is worth mentioning that though the present study includes many countries related to different continents, there is still a need for a study that would cover more variables. This research is thus a call for more ideas and efforts to focus on various other aspects of online education.

## Data availability statement

The original contributions presented in the study are included in the article/Supplementary material, further inquiries can be directed to the corresponding author.

## Ethics statement

The studies involving human participants were reviewed and approved by Ethics Committee of the University of Hradec Kralove no2/2021. The patients/participants provided their written informed consent to participate in this study.

## Author contributions

MP and BK: concept of the research, research design, and data collection. LA-O: data collection. AC-E and SD: statistics. All authors contributed to the article and approved the submitted version.

## Funding

This project is part of Excellence 2022 run at the Faculty of Informatics and Management at the University of Hradec Kralove, Czech Republic.

## Conflict of interest

The authors declare that the research was conducted in the absence of any commercial or financial relationships that could be construed as a potential conflict of interest.

## Publisher’s note

All claims expressed in this article are solely those of the authors and do not necessarily represent those of their affiliated organizations, or those of the publisher, the editors and the reviewers. Any product that may be evaluated in this article, or claim that may be made by its manufacturer, is not guaranteed or endorsed by the publisher.
